# Using Deep Learning to Recognize Therapeutic Effects of Music Based on Emotions

**DOI:** 10.3390/s23020986

**Published:** 2023-01-14

**Authors:** Horia Alexandru Modran, Tinashe Chamunorwa, Doru Ursuțiu, Cornel Samoilă, Horia Hedeșiu

**Affiliations:** 1 Faculty of Electrical Engineering and Computer Science, Transilvania University of Brasov, 500036 Brasov, Romania; 2 Romanian Academy of Scientists, 050044 Bucharest, Romania; 3 Romanian Academy of Technical Sciences, 010413 Bucharest, Romania; 4 Electrical Machines and Drives Department, Technical University of Cluj Napoca, 400027 Cluj-Napoca, Romania

**Keywords:** music therapy, artificial intelligence, deep learning, neural networks, python

## Abstract

Music is important in everyday life, and music therapy can help treat a variety of health issues. Music listening is a technique used by music therapists in various clinical treatments. As a result, music therapists must have an intelligent system at their disposal to assist and support them in selecting the most appropriate music for each patient. Previous research has not thoroughly addressed the relationship between music features and their effects on patients. The current paper focuses on identifying and predicting whether music has therapeutic benefits. A machine learning model is developed, using a multi-class neural network to classify emotions into four categories and then predict the output. The neural network developed has three layers: (i) an input layer with multiple features; (ii) a deep connected hidden layer; (iii) an output layer. K-Fold Cross Validation was used to assess the estimator. The experiment aims to create a machine-learning model that can predict whether a specific song has therapeutic effects on a specific person. The model considers a person’s musical and emotional characteristics but is also trained to consider solfeggio frequencies. During the training phase, a subset of the Million Dataset is used. The user selects their favorite type of music and their current mood to allow the model to make a prediction. If the selected song is inappropriate, the application, using Machine Learning, recommends another type of music that may be useful for that specific user. An ongoing study is underway to validate the Machine Learning model. The developed system has been tested on many individuals. Because it achieved very good performance indicators, the proposed solution can be used by music therapists or even patients to select the appropriate song for their treatment.

## 1. Introduction

Counseling, music therapy, physical exercise, and other practices can help an individual’s mental health. Music, on the other hand, is essential in everyday life. Music could both cause and stabilize mood swings [1].

Studies have shown that listening to music has the benefits of lowering heart rate and stress hormone levels [2]; thus, it has been used to reduce stress and anxiety for many years. Although music has long been used in therapy, few intelligent/smart applications can assist and support the medical therapist in selecting the most appropriate songs for his patients. A. Raglio et al. [3] used machine learning methods to identify the main predictors of the relaxation effects of listening to music. The authors assigned approximately 300 participants to listen to random music for 9 min. However, the process was not automated because relaxation levels were recorded before and after the listening experience using a Visual Analog Scale (VAS). The effect music listening had on relaxation was therefore predicted using a decision tree.

Listening to music is a technique used by music therapists in a variety of clinical treatments [4,5]. Research conducted on the importance of listening to music shows that many health problems can be treated using music therapy. An experimental study in the field of listening to music, chosen by the participants after stress exposure, has discovered that it improves mental balance [6]. According to recent surveys conducted by music therapists from various countries, a percentage of 42.7% of practitioners in this field use music in the therapy they provide to their patients [7].

Recent advances in Artificial Intelligence and Machine Learning, particularly since 2010, have enriched the signal processing landscape by providing new tools for signal estimation, classification, prediction, and manipulation. This not only resulted in significant performance gains in various long-standing problem domains (e.g., speech, computer vision, medical diagnosis), but it also enabled the development of new classes of nonlinear functions. Open questions in fundamental signal processing include robustness, adaptivity, and performance analysis. According to B. Sandler [8], incorporating new techniques into emerging architectures will most likely result in new system-level solutions for a variety of applications, leveraging their strengths while overcoming inherent weaknesses.

Music has been shown to have physical and mental health benefits, including improved cardiovascular health, is strongly connected to reducing cases of dementia in older populations, and improves markers of general mental well-being, such as stress reduction. A UK-based research team used a spectrum scale of anxious/negative to calm/positive to assess the effects of various musical pieces on listeners [9]. They gathered the initial data from listener reports, proven to be a reliable predictor of emotional response. They later fed this information into a supervised machine learning algorithm, which predicted additional types of music with strong affective properties. Testing the new music led to a feedback loop that showed that the ML system is an efficient way of identifying songs with desired effects. They analyzed specific data from MIDI files—such as pitch, melody, timing, and dynamics—and correlated each characteristic with the scores on the positivity scale.

Previous research has not thoroughly addressed the relationship between music features and their therapeutic effects on patients using the appropriate audio signal processing. In the current experiment, the dominant emotion conveyed by a specific musical sequence was chosen using an Artificial Intelligence model. The basic emotion wheel [10] describes the types of emotions into which songs are classified. A categorical approach was used, with the music divided into groups, and each group was described with an adjective (e.g., -sad, happy, boring, etc.). The experiment aimed to create a Machine Learning model that could predict whether a specific song has therapeutic effects on a specific person.

Aside from the introduction, this paper is divided into four sections. The second presents related studies, the third describes the material and methods used in the current study, the fourth focuses on presenting the results, and the final one describes the conclusions.

## 2. Related Works

B. Manaris et al. [11] presented the findings of an ongoing project about music information retrieval and music psychology. Their study investigated power law values for musical information retrieval. Power laws are statistical patterns with large proportions displayed by various natural and man-made phenomena. They propose Armonique, a prototype music search engine that uses power law values to capture both melodic and timbral characteristics of music. The user enters a piece of music as input. The engine searches the database for songs similar to this one, comparing the characteristics of the songs. The system includes a database of 9153 tracks from various genres such as Baroque, Classical, Romantic, Impressionist, Modern, Jazz, Country, and Rock. This data set was originally encoded using MIDI format, which helped in the extraction of melodic features, and later was converted to MP3 for timbre feature extraction. Pitch, chromatic tone, duration, the timeframe between repeated notes, the timeframe between repeated durations, melodic and harmonic intervals, melodic and harmonic consonance, melodic and harmonic bigrams, chords, etc., were all defined by the authors. Power-law values appear to correlate with aspects of human emotions and aesthetics, which suggests they hold great promise for content-based music querying and retrieval. Extraction and classification of power law features can lead to novel technological applications for information retrieval, knowledge discovery, and digital library navigation [11]. However, the achieved accuracy of around 76% can be improved.

The EMOPIA dataset, a shared multimodal database used for detecting emotions in Pop Piano music, is presented by the authors of [12]. Their dataset includes 1.087 annotated music clips from 387 songs. Creating the dataset includes song list curation, clip selection, and emotion annotation. The authors used various MIDI-based features and examined the distributions over the four quadrants of emotion to observe the emotional correlation of the musical attributes in EMOPIA. The characteristics used in this study were note density, length, velocity, and key distribution. The proposed model performed well in both four-quadrant and valence-wise emotion classification. In another study, H. Lee et al. [13] examined the similarity between the ratings of nine categories of perceived moods in music. They estimated their alignment with four popular mood detection algorithms by analyzing the responses of 166 participants from Brazil, South Korea, and the United States. The authors created a database of 360 pop songs from the abovementioned countries. They used Spotify’s Web API to run search queries for all unique songs, retrieving a maximum number of 50 results. According to this study, very simple mood attributes such as energetic, sad, cheerful, and boring are highly agreed upon by all listeners. Some of these properties (such as loudness and tempo) are low-level features that can be used by mood detection algorithms.

Handling the context is another critical aspect of effective mood prediction. The authors of [14] describe three approaches for dynamic emotion prediction based on Long Short-Term Memory (LSTM). The developed models were validated in real-time using a standard dataset annotated with arousal-valence values, and the authors chose the best-performer model. This study demonstrated that LSTM-based attention models outperform other transformers in terms of dynamic emotion prediction, as measured by the Kendall and R2 metrics. J. de Berardinis et al. [15] propose a new computational model that considers the role of different musical voices in predicting the emotions music elicits. The authors combine source separation algorithms for separating music signals into independent song elements to extract features and recognize emotions. EmoMucs has the advantage of providing insights into the relative contribution of different musical elements to the emotions perceived by listeners by using different fusion strategies and models trained independently.

The study from [16] shows that when it comes to mood classification, listening-based features outperform content-based ones because embeddings obtained through matrix factorization of listening data are more informative about a track’s mood than embeddings based on audio content. The authors used a subset of 67 k tracks from the Million Song Dataset and found that listening data outperformed audio-based embeddings in classifying moods in the proposed dataset.

Music is universally appreciated for the effects it produces. T. Greer et al. [17] researched three aspects of the complex human experience of listening to music: neural (how the brain responds to music), physiological (how the body responds to music), and emotional (how people report happiness or sadness during listening to a song). The authors employed a set of prediction models based on Multivariate Time Series (MTS), with audio signal characteristics serving as predictors. Previous research suggests that auditory features such as dynamics, timbre, harmony, rhythm, and register are related to emotion [18]. R. Delbouys et al. [19] investigated the task of multimodal music mood prediction based on an audio signal and track lyrics. The authors replicated the implementation of traditional feature engineering-based approaches and proposed a new deep learning-based model. They used a mel-spectrogram as the network’s input for audio, with 40 mel-filters and 1024 sample-long Hann windows with no overlapping at a sampling frequency of 44:1 kHz. Concerning the arousal detection task, the results show that this approach outperforms classical models.

Melody and lyrics, two distinct human cognitive abilities, are typically combined in music to convey emotions. L Xu et al. [20] investigated the effects of LIWC-based lyrical features on emotions conveyed by music using Linguistic Inquiry and Word Count (LIWC) technology to extract lyric features from 2372 Chinese songs. The proportion of words conveying negative emotions was inversely related to the perceived valence of music. In contrast to their utility in the emotion recognition model, lyrical features such as the frequency of use of words associated with sadness, as well as positive and neutral emotions, played an important role in the prediction model’s valence. Understanding the other party’s emotions is one of the key tasks associated with the implicit channel in human interaction. To tackle that task, R. Cowie et al. [21] examined basic issues in developing signal processing and analysis techniques, and, at the same time, the need to consolidate psychological and linguistic analyses of emotion, unlike previous approaches aimed at recognizing emotions using facial speech or gesture recognition.

Hoang et al. [22] see the potential of the contextual information from the scene. In their study, the general background data are also considered complementary cues for emotion prediction. The research of G. Ramet et al. [23] studied the use of attention mechanisms to enhance the performance of the state-of-the-art deep learning model in Speech Emotion Recognition. They introduced a new Long Short-Term Memory (LSTM)-based neural network attention model that achieved more than 68% weighted accuracy on 4 classes, using 5-fold cross-validation.

## 3. Materials and Methods

This section describes the materials and methods used in the current paper’s experiment. The goal was to create a Machine Learning model that can predict whether a specific song has therapeutic effects on a specific person. The model will consider a person’s musical and emotional preferences, as well as the previously mentioned aspects in terms of frequencies.

**Figure 1:**
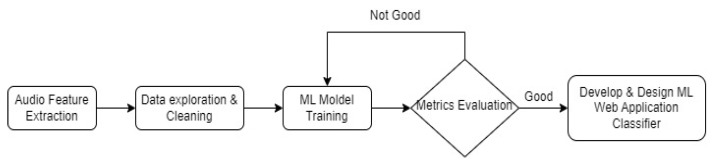
Experiment Pipeline.

The full pipeline of the experiment is the following (Figure 1):

- Extract audio features;
- Exploratory Data Analysis;
- Clean Dataset;
- Train Initial Machine Learning Model;
- Evaluate metrics of the Model;
- Design & Develop Machine Learning Classifier.

Because everything has a vibration, music therapy and sound healing focus on specific frequencies. Six of these frequencies, known as solfeggio frequencies, are specific tones known since Antiquity to have a beneficial effect on the mind and body. They were used in various rituals and ceremonies from ancient India to medieval Europe. Researchers have only recently begun to solve the mystery surrounding these frequencies, including 528 Hz, a tone that has gained attention for its power of healing and emotional release [24].

Solfeggio frequencies became popular again in the 1970s. The six important frequencies thought to raise vibrations generating therapeutical effects and helping heal are the following [25,26]:

- 96 Hz—helps people eliminate feelings such as fear, guilt, and grief;
- 432 Hz—clears negativity and triggers a positive change. [26] shows that music tuned to 432 Hz slows down the heart rate compared to 440 Hz;
- 528 Hz—one of the most important (also known as “love frequency”) is the frequency of transformation and DNA repair, and also helps to increase awareness;
- 639 Hz—helps reinforce relationships and connections and increases empathy and harmony;
- 741 Hz—known as a detoxifying frequency, it also helps solve problems;
- 852 Hz—beneficial for spiritual self-fulfillment.

K. Akimoto et al. [24] discovered that music tuned to the frequency of 528 Hz significantly reduced stress after only a few minutes of listening. Another study [27] found that 528 Hz reduced the toxic effects of ethanol, which is the main ingredient in alcoholic beverages. Furthermore, the authors observed that this frequency increased cell life by about 20%.

### 3.1. Audio Feature Extraction Data Exploration and Cleaning

A typical audio processing process involves acquiring data and extracting acoustic features relevant to the problem, followed by decision-making schemes involving detection, classification, and knowledge fusion.

The musical features used in this experiment are the following:

1. Spectral characteristics:
  - Spectral centroid—the mean frequency of the signal weighted by the magnitude;
  - Spectral roll-off—how many frequencies are concentrated below a certain threshold;
  - Spectral flux—how much the frequency varies over time;
  - Mel Frequency Cepstral Coefficients (MFCCs).
2. Temporal characteristics:
  - Zero-crossing rate—the number of time domain crossings in a frame;
  - Temporal centroid;
  - Log attack time—the time required to reach the maximum amplitude of a signal from a minimum time threshold.
3. Melodic/harmonic characteristics:
  - Tone class profile;
  - The clarity of the key;
  - Harmonic change;
  - Musical module.
4. Rhythmic characteristics:
  - Beat histogram (measured in beats per minute);
  - Medium tempo.

In this experiment, the Python library Librosa was used [28]. This library includes several methods for signal processing and extraction, such as spectral and rhythmic features.

The Million Song dataset was used for this experiment. This collection is a free collection of audio features and metadata for one million pieces of contemporary popular music [29].

**Figure 2:**
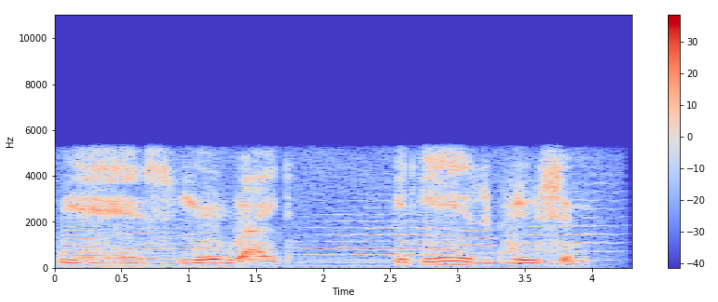
Spectrum of frequencies of signal (Spectrogram).

The extracted audio features can be seen on a spectrogram, which depicts the frequency spectrum of a signal as it changes over time. Figure 2 depicts a song’s spectrogram as a heat map, with the intensity shown by varying color gradients.

The Python script saves all computed features to a CSV file. The CSV contains one column for each of the following characteristics: tempo (in beats per minute), root-mean-square (RMS), chronogram, mel-spectrogram, spectral centroid, spectral contrast, spectral roll-off, zero-crossing rate, harmonizing, and Mel-frequency cepstral coefficients (MFCCs).

**Figure 3:**
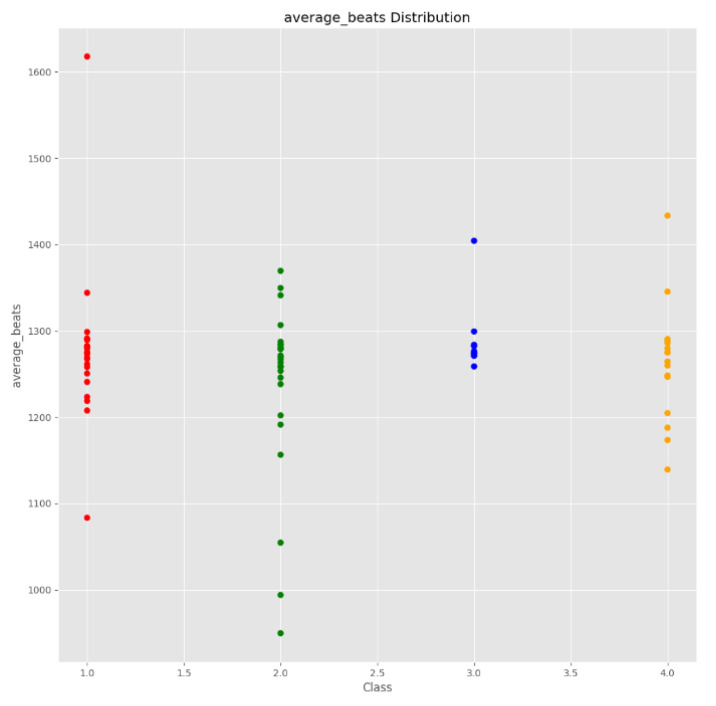
Audio features file.

The exploratory data analysis step was performed after extracting the audio features from the dataset. The main goal of this step was to determine which characteristics could be used as strong indicators to make an accurate prediction. The distribution of each variable was graphically plotted and then analyzed (as shown in Figure 3 for average beats).

The most relevant 10 audio features were selected after analyzing each feature, and the others were removed from the dataset.

Because labeled data is used in classification problems, these labels were applied to each audio file. The songs were divided into four categories, each represented by a different color: “energetic”, “calm”, “happy”, and “sad”. These categories were chosen based on M. Nuzzolo’s [30] article, which explains the best way to categorize music by mood.

### 3.2. Training the Machine Learning Model

A MinMaxScaler was used in the feature normalization process to ensure that all values between 0 and 1 were preserved, while also preserving the original shape of the data [31]. Finally, the dataset was divided, with 80% designated for training and 20% for testing.

**Figure 4:**
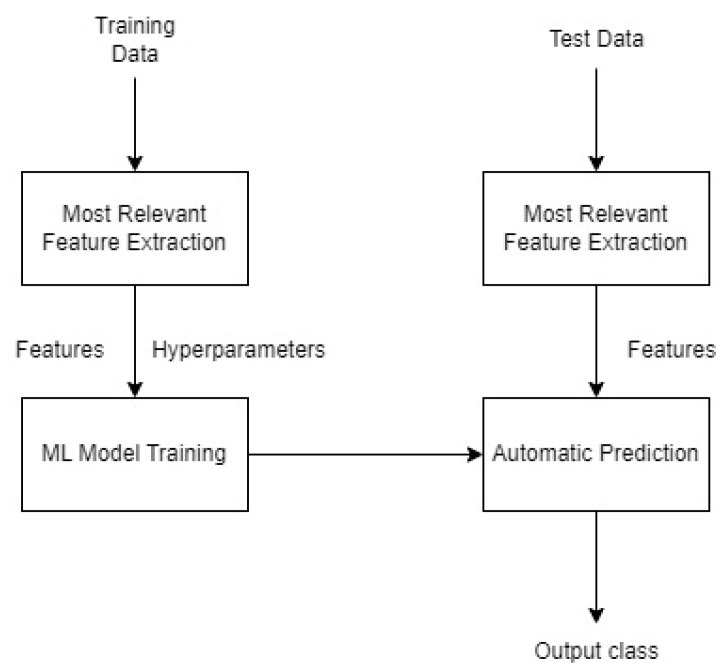
ML Model Development Pipeline.

Figure 4 depicts the entire pipeline and steps for developing the Machine Learning model.

The model was built using the Keras library, designed to allow the rapid development of deep neural networks [32]. Because the main goal is to categorize songs into four mood categories (happy, sad, energetic, or calm), the ML model is a multi-class neural network. In this experiment, a KerasClassifier with a ReLU (Rectified Linear Unit) activation function was used.

**Figure 5:**
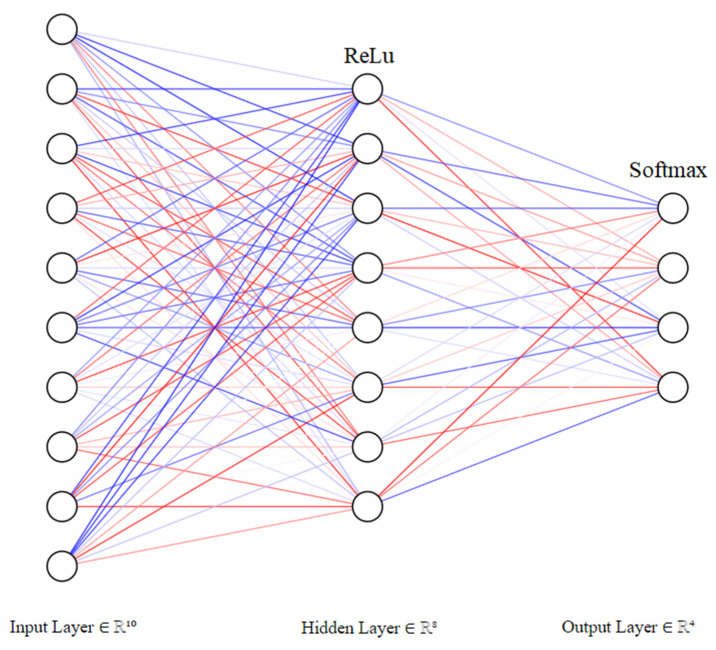
Neural Network Architecture.

Several possible values were tested and compared when selecting the input and output layers, as well as the activation functions. The layers of the developed neural network are as follows (Figure 5):

- An input layer with 10 audio features as input;
- A deeply connected hidden layer with multiple nodes with a Rectified Linear Unit (ReLU) activation function;
- An output layer containing four outputs (one for each category) with a Softwax activation function. Therefore, a classifier with an estimator role was also needed.

The estimator was evaluated using K-Fold Cross Validation. After experimenting with various possible values, the number of splits was set to K = 10. The model’s overall accuracy was 91.49%. The model was trained on 8000 samples in the current experiment.

### 3.3. Evaluating the Model

**Figure 6:**
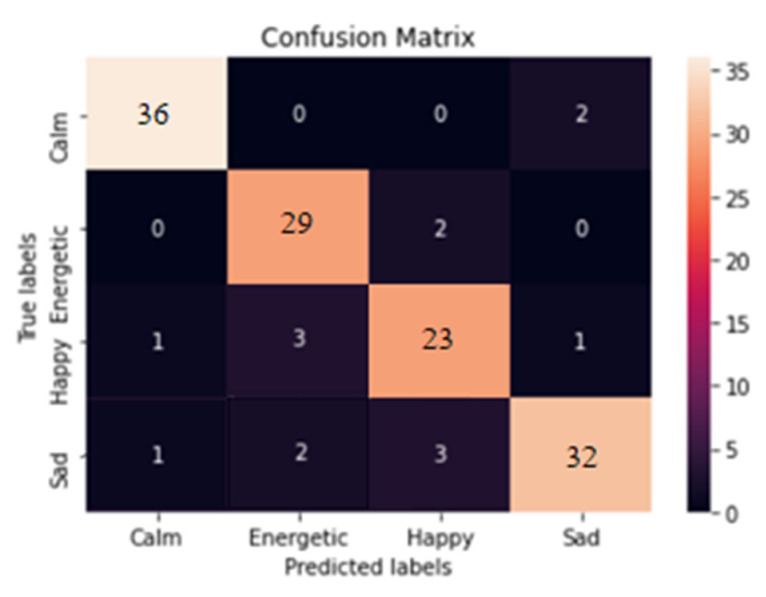
Confusion Matrix.

A confusion matrix was plotted using the Seaborn Library and Matplotlib to examine the model’s performance in detail (Figure 6). The accuracy of the model was also calculated.

With a final accuracy score of 94% and an examination of the Confusion Matrix, the model classified calm, happy, and energetic songs very well, but the accuracy for sad songs was slightly lower at 85%. It also attempted to improve the model’s accuracy by modifying some parameters, such as batch size, number of epochs, and the aggregation or deletion of some features used to train the model.

Deep Learning Algorithms can be used to implement ideas or projects involving the automation of tasks that require a significant amount of time to interpret. They can also assist in learning more about the world of data science and music taste trends.

Finally, a dedicated function was developed to predict the state conveyed by a song passed as a parameter using the previously created neural network.

### 3.4. Web Application for Classification

The classification is based on a dataset whose features have already been extracted by the algorithm presented in Section 3.3, using a web application developed in HTML5 with JavaScript. To perform the classification, the files containing the extracted features generated by the Python application must be provided in either CSV or JSON format. The data is also validated and, if necessary, normalized to ensure that the algorithm performs optimally.

The classification algorithm’s default parameters are defined in the JavaScript file, but they can be changed by the user via the web application’s user interface (UI). The following parameters had default values: number of epochs, learning rate, test data set size, number of hidden units, and activation function for both hidden and output layers.

These parameters are set to the values shown in Table 1 by default.

**Figure 7:**
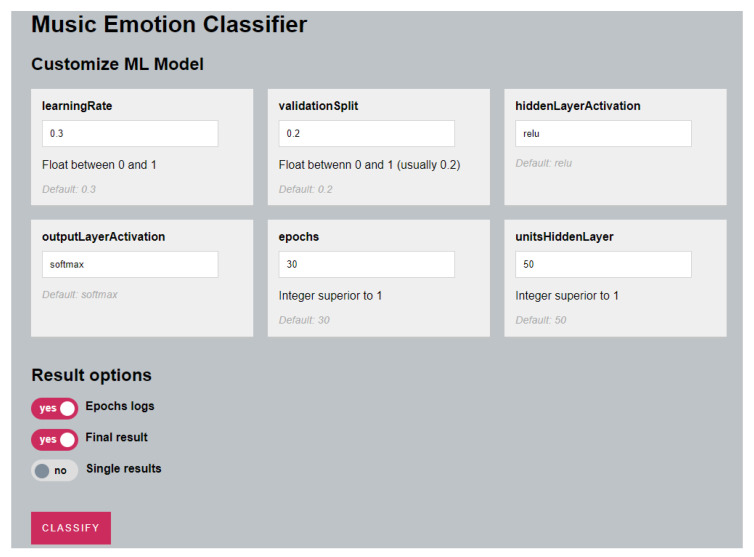
Model Training Web Application UI.

The user interface used to configure the parameters is illustrated in Figure 7.

The songs are categorized when one presses the Classify button, and the results are displayed in the browser’s console. When the classifier is run, the performance parameters for each step are displayed. The accuracy and loss improved with each run, reaching a final accuracy value of more than 90% after 120 epochs.

Another web application was developed, allowing the music therapists to select (i) some characteristics for the patient, (ii) a song, and, based on the trained Machine Learning Model described above, the app will be able to indicate whether that specific song will have a therapeutical effect for the patient. 

**Figure 8:**
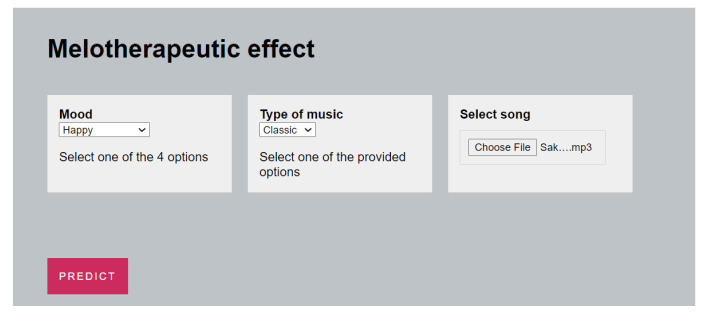
ClassificationWeb Application UI.

The application asks the patient about his favorite type of music and his current mood before making a prediction. If the selected song is inappropriate, the application, using Machine Learning, will recommend another type of music that may be useful for that user. Figure 8 depicts the User Interface of the Application.

## 4. Results and Discussions

This section summarizes the specific findings of this article and suggests opportunities and recommendations for further research. The research was carried out with the assistance of the Competence Valorization and Transfer Center (CVTC) from the Transylvania University of Brasov-Romania, in partnership with the Faculty of Electrical Engineering and the Faculty of Music at Transilvania University.

Although the developed Machine Learning model performed well in both the training and evaluation phases, it is critical to test and validate with people who are willing to use therapy as a solution to various problems. An ongoing study is being conducted to validate the Machine Learning solution, and the developed system has already been tested on a large number of people. The participants were either CVCT members or Music Therapy Master Program students, who all had different moods and musical tastes. They signed an ERB agreement to comply with the General Data Protection Regulation (GDPR). These subjects used the application on various days and in various moods, and the Machine Learning model correctly predicted and chosen in approximately 91.6% of cases.

Because previous studies have not thoroughly addressed the relationship between music features and their therapeutical effects via audio signal processing, this paper proposed a Machine Learning solution for recognizing the therapeutic effect conveyed by music. As a classifier, the algorithm described in this paper employs a multi-class neural network. It comprises an input layer with ten features, a deeply connected hidden layer with multiple hidden units, and an output layer. A web application for customizing the hyperparameters for the machine learning model, as well as another application for predicting whether a song is suitable for a specific person, was also developed. 

**Figure 9:**
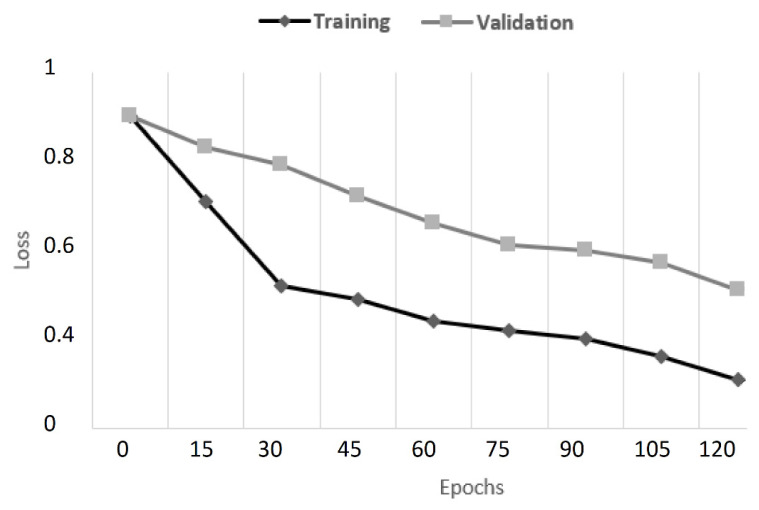
Performance indicators.

Figure 9 depicts the train and validation loss values over all epochs. Furthermore, the model had 89% precision, 91% recall, and an F1-score of 0.899.

This current experiment, however, can be improved. The proposed solution has the limitation that users must select their current mood, which is somewhat subjective. If users are unsure about which mood to choose, one solution to improving the current application could be to allow them to select multiple options. In addition, future enhancements will use edge AI methods for data processing, allowing the Machine Learning Model to be deployed directly on the PSoC6 microcontroller device. The ModusToolbox environment allows one to create and run pre-trained machine-learning models on the PSoC6 directly. The model is compatible with this microcontroller and can be, technically, deployed on any IoT device because it was created using the Keras library in Python. By doing so, the entire system becomes portable and can be controlled via Bluetooth or Wi-Fi if an internet connection is available.

## 5. Conclusions

Music listening has long been used in clinical treatments by music therapists. As studies on the importance of listening to music have been conducted, and many health problems can be remedied with the help of music therapy, it is critical for practitioners or even patients to use an intelligent system for selecting the right music for each patient.

Because previous studies have not addressed the relationship between music features and their therapeutic effects on patients using audio signal processing, this paper attempts to address this issue. The experiment aimed to create a Machine-Learning model that could predict whether a specific song has therapeutic effects on a specific person. The model was trained to consider the solfeggio frequencies as well as the characteristics of a specific person in terms of music and emotions. A section of the freely available Million Dataset was used to train the machine learning model.

According to Section 3, the model achieved very good performance indicators and an overall accuracy of more than 94%. There is also an ongoing validation process for people who want to use music therapy to treat their problems. So far, it has been tested on several people with positive results. As a result, the proposed solution can be used by therapists and others who want to benefit from the therapeutic effects of music.

## Figures and Tables

**Table 1:** Default parameter values.

Parameter	Value
epochs	30
validation split	0.2
learning rate	0.3
hidden units	50
hidden layer activation function	ReLU
output layer activation function	Softmax

## Data Availability

The data presented in this study are available on request from the corresponding author.
